# An Imaging and Histological Study on Intrahepatic Microvascular Passage of Contrast Materials in Rat Liver

**DOI:** 10.1155/2017/1419545

**Published:** 2017-02-15

**Authors:** Qian Xia, Yuanbo Feng, Ting Yin, Yewei Liu, Guozhi Zhang, Jianjun Liu, Linjun Tong, Robin Willemyns, Jie Yu, Raymond Oyen, Gang Huang, Yicheng Ni

**Affiliations:** ^1^Department of Nuclear Medicine, Renji Hospital, School of Medicine, Shanghai Jiao Tong University, 160 Pu Jian Road, Shanghai 200127, China; ^2^Department of Imaging and Pathology, University Hospitals, KU Leuven, Herestraat 49, 3000 Leuven, Belgium

## Abstract

*Background*. Lipiodol has been applied for decades in transarterial chemoembolization to treat liver malignancies, but its intrahepatic pathway through arterioportal shunt (APS) in the liver has not been histologically revealed. This rodent experiment was conducted to provide evidence for the pathway of Lipiodol delivered through the hepatic artery (HA) but found in the portal vein (PV) and to elucidate the observed unidirectional APS.* Methods*. Thirty rats were divided into 5 groups receiving systemic or local arterial infusion of red-stained iodized oil (RIO) or its hydrosoluble substitute barium sulfate suspension (BSS), or infusion of BSS via the PV, monitored by real-time digital radiography. Histomorphology of serial frozen and paraffin sections was performed and quantified.* Results*. After HA infusion, RIO and BSS appeared extensively in PV lumens with peribiliary vascular plexus (PVP) identified as the responsible anastomotic channel. After PV infusion, BSS appeared predominantly in the PV and surrounding sinusoids and to a much lesser extent in the PVP and HA (*P* < 0.001). Fluid mechanics well explains the one-way-valve phenomenon of APS.* Conclusions*. Intravascularly injected rat livers provide histomorphologic evidences: (1) the PVP exists in between the HA and PV, which is responsible to the APS of Lipiodol; and (2) the intrahepatic vascular inflow appears HA-PVP-PV unidirectional without a physical one-way valve, which can be postulated by the fluid mechanics.

## 1. Introduction

Lipiodol (LPD) as a medicine has been existing for over 100 years, yet it is still in active clinical use under a trademark of LIPIODOL® in the USA and LIPIODOL ULTRA FLUID® outside the USA [1]. LPD was first synthesized in 1901 by French pharmacist Marcel Guerbet (1861–1938) and was initially marketed for therapeutic purposes on clinical indications covering pulmonary and cardiovascular diseases (e.g., asthma, angina, and pericarditis), impetigo, rheumatism, syphilis, and goiter prophylaxis [1]. Since 1921 following the discovery of X-ray by Wilhem Roentgen, the radiopaque property of LPD has been exploited for radiological diagnoses in lymphangiography, hysterosalpingography, sialography, and fistulography [1].

LPD is an oily contrast medium consisting of a mixture of long-chain (C16 and C18) di-iodinated ethyl esters of fatty acids from poppy seed oil, which contains 98% unsaturated fatty acids [1, 2]. Thus, intravascular administration of LPD was considered a contraindication due to possible fatal embolic complications. However, since the early 1980s, LPD has been applied for transcatheter arterial chemoembolization (TACE) of primary and metastatic liver cancers, taking advantages of the unique liver anatomy and the blood supply to the malignancies, which symbolizes a major step forward and the dawn of a new life for this venerable drug [3–7]. After a huge number of preclinical and clinical studies over the last 35 years, the benefits associated with the use of LPD for TACE have been widely acknowledged with continuing interests in this field. Likewise, the versatile properties of LPD as a tumor-seeking, drug-delivery, embolic and radiopaque agent are also extended to other clinical indications for chemoembolization procedures beyond hepatic malignancies [1, 3–7].

In the context of TACE, LPD that was delivered via the hepatic artery (HA) route could be almost always found in the portal vein (PV) of the nontumorous liver [7–9]. Several hypotheses have been put forward to explain this phenomenon. The liver is known to differ from other organs by having a dual vascular inflow system to get its blood supply via both the HA and PV. After arterial administration in TACE procedure, LPD could be revealed in the PV of both the patients and animals [7–10]. By using scanning electron microscopy and in vivo microscopy, Kan and Madoff reported the passage of LPD through peribiliary vascular plexus (PVP) between the terminal hepatic arterioles and the terminal portal venules [8]. More recently, by using micro-CT imaging in rats with injection of radiopaque Microfil® into the HA and/or the PV, Kline et al. observed unidirectional hepatic arterioportal shunt (APS) occurring between the HA and PV branches with an assumed one-way-valve-like mechanism [11].

**Figure 1:**
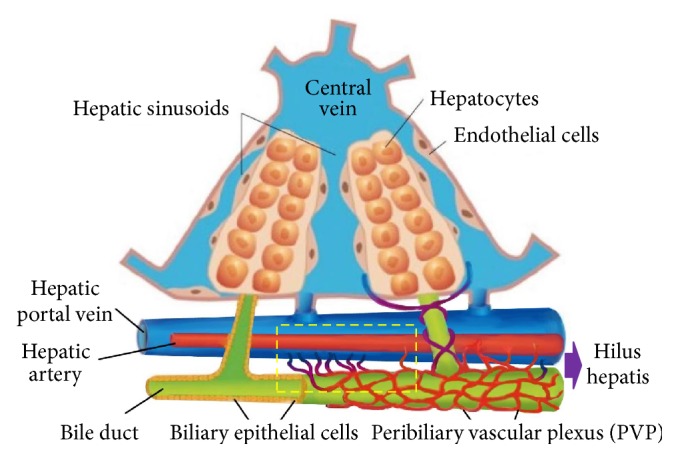
A diagram of schematic microanatomy of partial hepatic unit modified from a figure of citation [12]. The dashed frame denotes the microcirculatory HA-PVP-PV structure in the portal tract that this study was intended to elucidate.

However, due to the limitations in experimental design and technical constraints, so far no direct histological evidence, as a gold-standard proof, for intrahepatic microvascular APS passage has been visually demonstrated [8, 11]. Furthermore, a sketch in the recent literature indicated the HA as the origin of the PVP but failed to display its draining connections to PV branches [12], likely due to a lack of hard evidence. Similar problem can be found in Figure 1 of a review article [13]. Therefore, all these support that clear and direct proofs as illustrated in Figure 1 of this paper are needed, which prompted us to conduct this experimental research.

We designed the present morphological study in rats by combining intravascular injections of multifunctional contrast materials, “real-time” digital radiography (DR) for microangiogram [14], histomorphology of cryotome and paraffin sections with special and standard stains, and mathematical assumptions in an attempt to experimentally elucidate the pathways of LPD trafficking through the hepatic microcirculation. The findings would also contribute to current understanding of intrahepatic anatomy and hemodynamics.

## 2. Materials and Methods

**Figure 2:**
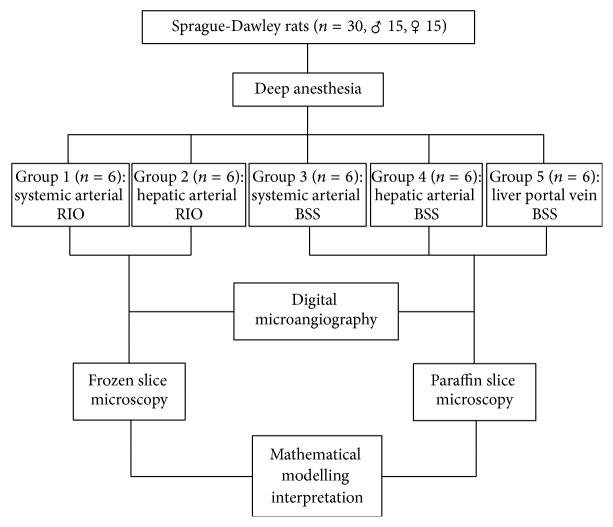
A flow chart of the experimental design.* Note*. *n*, number; ♂, male; ♀, female; RIO, red iodized oil; BSS, barium sulfate suspension.

This rodent experiment was approved by the institutional ethical committee for animal research and welfare and performed according to a designed protocol (Figure 2).

### 2.1. Preparation and Application of Two Multifunctional Contrast Agents

A custom-made multifunctional contrast dye, namely, red iodized oil (RIO), was formulated by solubilizing 500 mg Oil-red-O pigment (Sigma-Aldrich) in 100 mL of Lipiodol (LPD) ultra-fluid (Guerbet, France) [15]. Being otherwise almost colorless as seen with LPD, RIO becomes a bright red dye for staining tissue structures especially blood vessels, making them discernable both grossly in organs/tissues and microscopically on frozen sections [15, 16]. Meanwhile, RIO functions as a contrast agent for both X-ray based imaging modalities and magnetic resonance imaging (MRI) without significantly altering physiochemical properties of LPD [15].

Barium sulfate suspension (BSS), such as Micropaque® (Guerbet, France), is a hydrophilic colloidal suspension with particular sizes ranging from 0.1 to 0.5 *μ*m in diameter [17]. Like LPD or RIO, BSS can pass through the blood vessels of any dimensions in animals. Being a hydrophilic alternative to the lipophilic LPD or RIO, BSS was also applied in this study as a contrast agent for microangiography and as a green-yellowish dye to trace vascular lumens on standard HE histological preparations of being affected with fewer artifacts.

### 2.2. Preparation of Animals and Study Design

Thirty Sprague-Dawley rats of equal gender weighing about 350 g were deep anesthetized with intraperitoneal pentobarbital (Nembutal; Sanofi Sante Animale, Brussels, Belgium) at a dose of 50 mg/kg and divided into five groups (*n* = 6 each) as seen with the study design (Figure 2).

**Figure 3:**
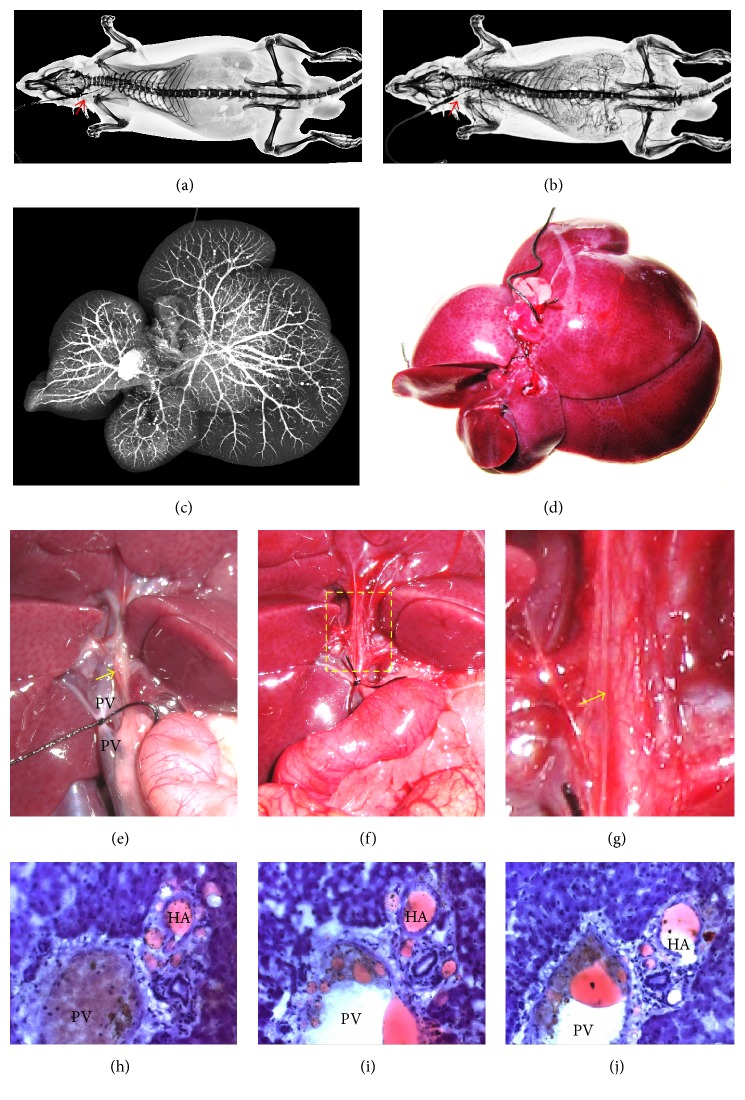
Whole-body DR, liver microangiography, and macro- and microscopy in rats. (a) Plain DR of a whole-body rat with its carotid artery catheterized (arrow); (b) after the stepwise infusion of RIO through the carotid artery (arrow), the entire arterial network was visualized, but the angiographic details of the liver were masked due to overlapping with bony thorax; (c) the DR images from the excised liver of a rat after systemic infusion of RIO revealed the entire hepatic vascular tree that was mainly composed of intrahepatic PV branches. RIO often caused beaded-stream appearance in larger vessels due to its oily droplets; (d) the corresponding liver specimen; (e) macroscopic view of the* hilus hepatis* region shows the CBD (arrow) and the ligated PV to prevent the drainage from other abdominal organs; (f) infusion of RIO through the abdominal aorta stained the vessels and liver in red; (g) more magnified view of the dashed region from (f) revealed the red PVP on the surface of CBD; (h, i, j) photomicrographs from serial frozen sections on a portal triad demonstrate the gradual transition of RIO from the HA cavity through the PVP capillaries to the PV cavity with some RIO appearing hepatic sinusoids (original magnification ×200).

*Group 1*. Systemic arterial infusion with RIO: right carotid artery was exposed and inserted retrograde with a 22 G infusion needle (Becton Dickinson SA, Madrid, Spain), while external jugular vein was cut open as a fluid exit. After euthanasia by overdose of Nembutal, heparinized normal saline was infused for rinsing blood circulatory system. Under radiographic monitoring (Figures 3(a) and 3(b)), RIO was infused till the abdominal vasculature was discerned. The entire liver was resected for further imaging and histological studies (Figures 3(c) and 3(d)).

*Group 2.* Infusion of RIO into the hepatic artery: to avoid the drainage from other visceral organs into portal vein, a plastic catheter was placed in abdominal aorta that was segmented by ligations between the diaphragm and superior mesenteric artery so that the injectant would mainly enter the HA. After ligation of the PV, RIO was intra-arterially injected until the liver, common bile duct (CBD), and gastroduodenal surface turned reddish (Figures 3(e)–3(g)). The liver was entirely resected for further imaging and histological studies. 

*Group 3.* Systemic arterial infusion with BSS: procedures were similar to Group 1 but water soluble BSS was injected with standard histologic protocol to better preserve liver morphology. 

**Figure 4:**
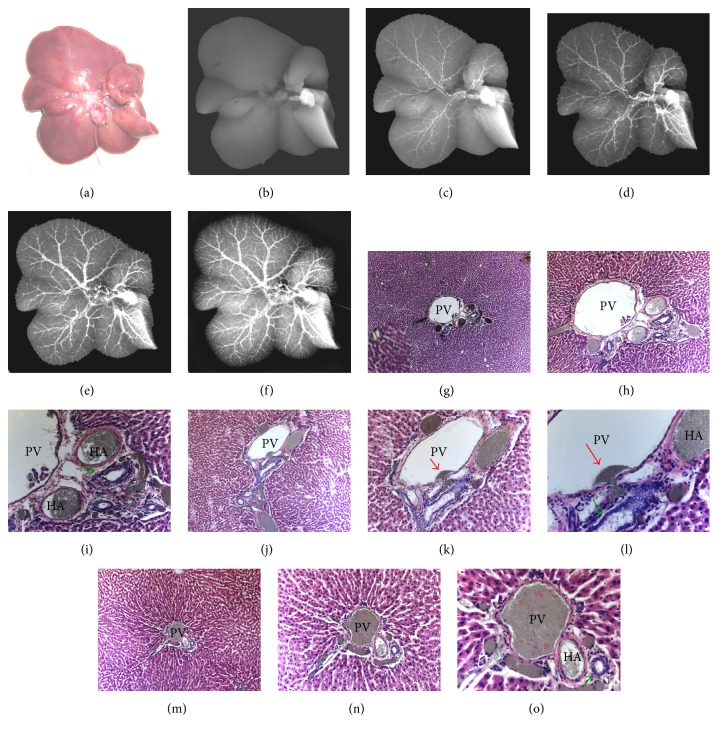
Stepwise hepatic arteriography and histomorphology from a rat liver. (a) An excised rat liver with the HA catheterized; (b) DR before contrast injection; (c, d, e, f) microangiograms after stepwise infusion of BSS into the HA display initial HA tree (c) followed gradually by predominance of the PV branches (f) without discernable HA-PV interconnections due to limited spatial resolution; (g, h, i) photomicrographs of a hematoxylin-eosin- (HE-) stained paraffin section focusing on a portal triad exhibit the cavities of the HA and PVP filled with yellow-greenish BSS granules that start to appear in the PV cavity but not in sinusoids and surrounding central veins (original magnifications of 50, 100, and 200); (j, k, l) microscopic HE-stained views of another portal triad from the same rat capture the moment when the BSS enters the PV cavity through a tiny anastomosis (arrow) from a PVP capillary (original magnifications of 100, 200, and 400); (m, n, o) photomicrographs of a HE-stained slice from the same group show full presence of BSS granules in the cavities of the PV and PVP despite having been injected through the HA with BSS starting to enter the surrounding sinusoids (original magnifications of 50, 100, and 400). Green arrow points the bile duct.

*Group 4.* Infusion of BSS into the HA in isolated liver: a polyethylene catheter of 0.6 mm in diameter (Intramedic®, NJ, USA) had been inserted retrograde into gastroduodenal artery, further entered into the HA, and anchored there before the entire liver was resected (Figure 4(a)). BSS was injected stepwise under the monitoring with DR (Figures 4(b)–4(f)) for better steering the amount of injected agent. 

**Figure 5:**
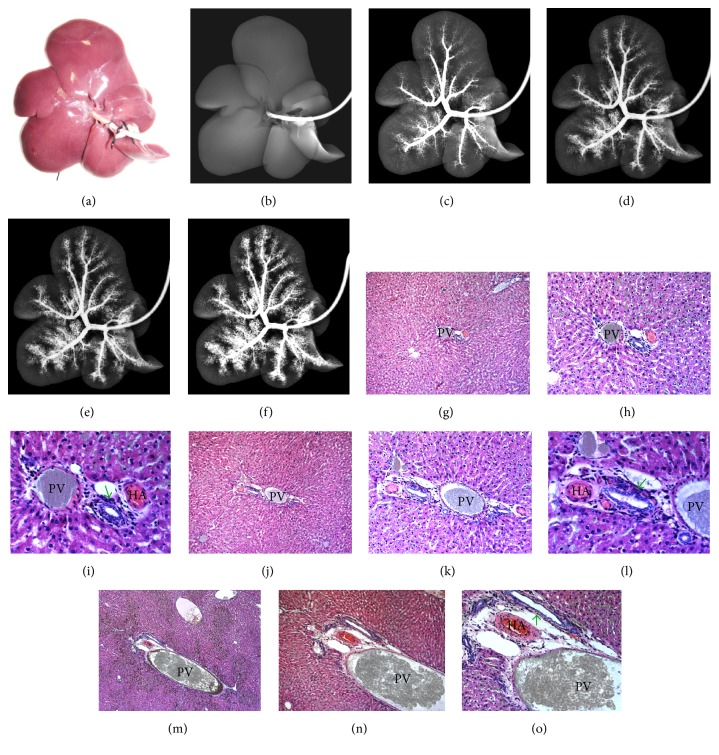
Stepwise hepatic portography and histomorphology from a rat liver. (a) An excised rat liver with the PV catheterized; (b) DR before contrast injection; (c, d, e, f) microangiograms after stepwise infusion of BSS into the PV display a gradual opacification of hepatic vascular tree solely composed of the PV branches with cloudy filling of the adjacent sinusoids but without discernable HA branches; (g, h, i) photomicrographs of a HE-stained paraffin slice focusing on a cross-sectional portal triad demonstrate the full presence of BSS granules in the PV cavity and the surrounding sinusoids but absence in the HA and PVP cavities (original magnifications of 50, 100, and 200); (j, k, l) microscopic HE-stained oblique-sectional views of a portal triad from the same group uncover the BSS particles mainly in the PV cavity and adjacent sinusoids (original magnifications of 100, 200, and 400); (m, n, o) HE-stained photomicrographs of another portal triad oblique section from the same group confirm full presence of BSS granules in the cavities of both the PV and sinusoids but obscurely in the HA and PVP cavities (original magnifications of 50, 100, and 200). Green arrow points bile duct.

*Group 5.* Infusion of BSS into the PV in isolated liver: a plastic catheter had been inserted into the PV and anchored by ligation before the entire liver was resected (Figure 5(a)). BSS was injected stepwise while being monitored by DR (Figures 5(b)–5(f)) for better steering the injected BSS.

### 2.3. Digital Radiography for Microangiography

The catheterized cadavers or livers from the rats were exposed by DR [14] using a Siemens Mammomat Inspiration system (Siemens, Erlangen, Germany) at 25 KV and 18 mA of X-ray before and after stepwise infusion of RIO or BSS and photographed by a digital camera.

### 2.4. Tissue Preparations and Histology

For visualizing RIO as a colored surrogate of LPD, modified tissue preparation was adapted. Tissue blocks were sampled from median, left, and right liver lobes and imbedded with OCT Tissue-Tek® compound (Sakura Finetek USA) onto a round cork plate and rapidly frozen to −30°C in dry ice. By using a cryotome (Microm HM 550, Walldorf, Germany), the frozen block was serially sectioned into 20 *μ* slices (at least 10 slices per block), which were thaw-mounted on glass slides. Since LPD was already stained red (RIO), only light staining of nuclei with alum haematoxylin was performed to minimize possible washout of RIO from the tissue particularly vascular lumen. With this technique, RIO could be found microscopically in relevant vessels depending on the injection route.

For visualizing BSS as a water soluble green-yellowish marker to study intrahepatic passage of LPD or RIO, liver tissues were fixed with 10% formalin and processed with paraffin sectioning into 5 *μ* slices with a microtome and hematoxylin-eosin staining for microscopic observation and digital photography (Axioskop, Zeiss, Germany). Morphological quantification was performed in groups where standard histology was applied. After randomly counting 100 portal triads per rat liver at ×100 magnification view, the presence or absence of BSS was rated as percentage in four different vascular lumens including the PV and HA branches, PVP, and surrounding hepatic sinusoids (Table 1). Microscopic observations on a large number (*n* = 600 per group) of portal triads allowed comprehension of dynamic distribution of BSS in the liver.

### 2.5. Mathematical Modelling

We proposed a hypothesis to mathematically explain the experimental phenomenon with the theory of fluid dynamics. Considering a local part of the blood vessel as a pipe of constant circular cross-section that is substantially longer than its diameter, the blood, with or without the administration of contrast materials as employed in this study, can be assumed to be an incompressible and Newtonian fluid in laminar flow, which satisfies a physical condition that is well described by Poiseuille's law [18]:

(1) $$\begin{array}{c} Q=\Delta P\cdot R^{-1}=\Delta P\frac{\pi r^{4}}{8\eta L}, \end{array}$$

where *Q* is the volumetric flow rate, Δ*P* the pressure difference, *R* the flow resistance, *r* radius of the vessel, *η* the dynamic viscosity, and *L* length of the pipe. This relationship shows a dominant influence of vessel radius on blood flow as compared to vessel length (*L*) or the blood viscosity (*η*). It can be seen that, with pressure difference (Δ*P*) being a must, the actual flow rate is very much governed by the magnitude of resistance (*R*), which is in turn determined by the radius (*r*). Thus, the pressure gradient Δ*P* is inversely related to *r*^4^ and any change in radius will result in an exponential change in the pressure gradient.

### 2.6. Statistical Analyses

Statistical analysis was performed using SPSS for Windows version 21. Chi-square test was utilized to semiquantitatively compare the BSS injected from hepatic artery and from portal vein with regard to the BSS deposition in the four different vascular lumens. A difference was considered statistically significant if the *P* value was less than 0.05.

## 3. Results

### 3.1. General Aspects

All experimental procedures were carried out according to the predesigned protocol (Figure 2) without accidental loss of animals. The clinical mammography unit was adapted well for DR-microangiography in rats. The newly formulated RIO and clinically available BSS worked well for histomorphology. Since the injected RIO or BSS equally passed the tiniest luminal structure of the PVP as being focused by this study, the outcomes could be crossly interpreted between these two contrast media; and since no difference was noticed between genders, morphologic results were pooled in 6 rats per group.

### 3.2. Microangiographic Findings

The stepwise infusion of RIO (Group 1) or BSS (Group 3) through the carotid artery caused gradual visualization of whole-body arterial network but without angiographic details of the liver due to overlapped bony thorax structures on perspective images (Figures 3(a) and 3(b)).

The DR images from the liver specimens from the rats after systemic infusion of RIO or BSS revealed the hepatic vascular tree that was mainly composed of intrahepatic PV branches (Figures 3(c) and 3(d)). RIO often caused beaded-stream appearance in larger vessels due to oily droplets of this agent (Figure 3(c)), whereas hydrophilic BSS showed continuous lines in the vasculature. All liver lobes were angiographically visualized. Portal veins, and less often hepatic veins, of the liver filled with the RIO or BSS were clearly shown, branching from the* hilus hepatis* to the edge of the liver. Hepatic arteries were considerably thinner distributing along the portal veins.

The arterial infusion of RIO (Group 2) stained the vessels (Figure 3(e)) in red (Figure 3(f)) and visualized the PVP on the surface of common bile duct (CBD) in a more magnified view (Figure 3(g)).

In Group 4 of the excised liver (Figure 4(a)) with stepwise infusion of BSS into the HA (Figures 4(b)–4(f)), the vascular tree was initially composed of only HA (Figures 4(c) and 4(d)) but soon the PV dominated (Figures 4(e) and 4(f)) without any visible linkages in between the HA and PV due to resolution limits. Sinusoids and hepatic vein were not seen due to imaging-monitored stepwise infusion.

In Group 5 of the excised liver (Figure 5(a)) with stepwise BSS infusion into the PV (Figures 5(b)–5(f)), the vascular tree was readily displayed with PV branches (Figures 5(c) and 5(d)), followed by cloudy opacification of the adjacent sinusoids (Figures 5(e) and 5(f)). HA branches or hepatic vein was unseen.

### 3.3. Histological Findings

Frozen and paraffin sections consistently depicted the intrahepatic microcirculatory pathways of RIO and BSS.

Being stained into red as RIO, the behavior of LPD could be traced microscopically on serial frozen sections (Figures 3(h)–3(j)). Focusing on portal triads, RIO appeared first in the hepatic arterial branches, irrigating the PVP capillaries (Figure 3(h)), and then entered adjacent PV branches through tiny anastomoses (Figures 3(i) and 3(j)).

After BSS infusion through the HA, all arterial branches were first filled with BSS that also entered PVP channels (Figures 4(g)–4(i)); the BSS granules from the PVP further arrived at the adjacent PV cavity through minute anastomosis (Figures 4(j)–4(l)); the PV branches could be completely filled with BSS, which then started to enter sinusoids (Figures 4(m)–4(o)).

After infusion through the PV, all of its branches were filled with BSS granules that also entered sinusoids but hardly appeared in HA branches and PVP channels (Figures 5(g)–5(o)). Under the controlled injection in this postmortem study, both RIO and BSS were almost never found in the central venules and hepatic veins (Figures 3–5).

The above-described microscopic observations are quantitatively summarized in Table 1. After HA injection, BSS granules could be found in almost all HA (98%) and PV (95%) branches as well as PVP cavities (97%), but in only a few adjacent sinusoids (20%), whereas, after PV injection, BSS granules appeared in almost all PV branches (99%) and surrounding sinusoids (90%), but in only about half of PVP channels (55%) and few HA branches (6%). There is no statistical difference between HA and PV injection routes in terms of BSS deposits in PV branches (*P* > 0.05). However, BSS distributions in HA, PVP, and sinusoid channels are all significantly different (*P* < 0.001) between the two injection routes.

### 3.4. Mathematical Modelling

The above microangiographic and histological findings confirm the presence of an intrahepatic HA-PV unidirectional shunt (or APS) as reported previously [11]. However, we failed to microscopically identify any one-way-valve-like structure as assumed by that study where the spatial resolution of the imaging technique was >50 *μ* [11]. Instead, we tend to attribute this phenomenon to (1) the fluid dynamics within the vascular systems of HA and PV branches (Figure 1) and (2) the presence of PVP channels of <50 *μ* in diameter as demonstrated in this study (Figures 3–5), which were yet undiscernible with X-ray based DR in this study or *μ*CT [11].

The observed unidirectional blood flow can be explained for both in vivo and postmortem scenarios upon a number of logical assumptions in order to fit Poiseuille's law: (1) the vessel length (*L*) and the viscosity (*η*) of the vessel content in Equation (1) are the same for the HA and PV; (2) the tiny PVP path between the HA and PV is always open and could be bidirectional; (3) empirically the radius of PV is about 10 times larger than that of the HA (e.g., 1 mm versus 0.1 mm); and (4) the elasticity of the wall can be considered similar in the HA and PV.

(*1) In the Case of In Vivo Condition.* One important physiological fact is that the blood pressure is substantially higher in HA than in PV, namely, about 100 mmHg and 15 mmHg in the HA and PV, respectively [11]. As such, the blood cross-flow between HA and PV through PVP channels is naturally driven by this HA-PV pressure gradient; that is, the cross-flow can only appear as a unidirectional flow. Therefore, intrahepatic arterially injected LPD, RIO, or BSS could easily make its way to the PV, but not in the opposite direction even if being infused through the PV. 

*(2) In the Case of Postmortem Condition.* In our postmortem experiments, the inherent blood pressure in a euthanized rat or excised liver vanished to 0 in both the HA and PV, while the only pressure in the system is caused by the injection. This can introduce a pressure gradient from HA to PV, or the way around, depending on through where the injection is conducted. The actual flow at the HA and PV “*intersection*” (PVP channels), however, is also affected by the resistance in each corresponding vessel. As given by Equation (1), the flow resistance would be 10,000 times higher in HA compared to PV, resulting from their 10 times difference in radius. Consequently, the flow with LPD, RIO, or BSS, injected from the HA, is much more likely to find its way toward the PV via PVP channels, where a substantially higher flow rate can be achieved. When injected from PV, however, the flow tends to remain in its current path, but the amount that goes into the HA is negligible due to its extremely strong resistance, which has been plausibly supported by our radiographic and microscopic observations (Figures 3–5, Table 1).

## 4. Discussion

Previous studies have shown that the hepatic arterial branches give rise to the vascular plexus around bile ducts within both extra- and intrahepatic portal tracts, which is called the peribiliary vascular or capillary plexus (PVP or PCP) [12, 13, 19, 20], which was omitted by other studies likely due to technical constraints [11, 21]. Human pathology has demonstrated alterations of the PVP under normal and diseased conditions [22], but it is impossible to expose the entry and exit of blood through this system. By staining red color to the liposoluble LPD [15] and using the BSS as a hydrosoluble alternative [14], this study has provided colorful histomorphologic evidence to support the previous “grayscale” findings [8] that indeed the PVP transported the arterially injected LPD and BSS into the PV branches. Although the PVP could be observed by the operator of the intravital microscope, the published black-white pictures failed to depict local structures, with labels or sketches needed to aid imagination [8, 23]. Thus the present study provides more demonstrative and dynamic proofs in this context.

We have also confirmed the puzzling unidirectional APS pathway in the liver as more recently reported [11]. But both the micro-CT applied there [11] and DR-microangiography used in this study proved insufficient to approach the structural details of this phenomenon due to insufficient spatial resolutions of maximum 50–100 *μ*m/pixel only [11, 24]. The diameters of the PVP connecting the HA to PV could range as small as ≤5 *μ* based on our microscopic observations, which is physically beyond the resolutions of X-ray based modalities. While being unable to microscopically identify the suspected presence of “one-way-valve” [11], upon intrahepatic anatomy, we tried to interpret this hemodynamic phenomenon by mathematical postulation of such a virtual “one-way valve” using the equation of fluid mechanics [18].

Because of the huge resistant discrepancy between the HA and PV resulting from their different radii and minute interconnecting PVP, injectants and blood tend to flow nearly unidirectionally from the HA to the PV, but not the other way round. Thus, this study might also impact on our current knowledge and practice in hepatology. Particularly these findings may modify the long-held assumption that the HA and PV branches run in parallel to each other within the liver and both vessels end up to the hepatic sinusoids to supply blood via their terminal branches. But, the more likely reality would be that arterial blood passes through the PVP and joins in the blood stream in the PV before irrigating the hepatic sinusoids as shown with our microangiographic and histologic findings, which also sounds plausible from an embryological point of view [25]. Further research deems necessary to assure that similarity exists between humans and rats in this aspect.

Clinically, LPD has been widely used in the TACE of hypervascular HCCs or metastatic tumors that are known to derive their blood supply almost exclusively from the HA [3–5]. One of the most important complications of TACE is hepatobiliary damage (HBD) with liver insufficiency, hepatic abscess, intrahepatic biloma formation, and so forth due to the occlusion of the PVP by LPD and/or the toxicity of the mixed anticancer drugs on vascular endothelial cells [26–31]. Thus, highly selective catheterization and adequate amount of regional LPD in the liver tumor with minimal amount to healthy hepatic tissue are crucial to HBD prevention [32], in which real-time imaging monitoring plays a key role for drug administration as suggested by this study.

Technically, experimental studies on LPD proved challenging, because oily LPD becomes void in both paraffin and conventional lipid staining methods due to dissolution and/or washout of LPD by the applied organic solvents. By the prior coloration with Oil-red-O [15] and modified staining method, for the first time to our knowledge, we could microscopically identify the distribution of LPD in various locations of intrahepatic microcirculation. Furthermore, our experiment showed that both RIO and BSS took the same route in hepatic vasculature, making it possible to replace LPD by BSS to conduct standard morphology and quantification.

Since we did not find any structural valves between the HA and PV, we proposed a hypothesis to explain this phenomenon by using the mathematical method of fluid dynamics. Based on Poiseuille's law, we find that the resistance appears at least 4 orders of magnitude stronger in the HA as compared to that in the PV, both in vivo and ex vivo, as a result of the difference in the radius of their lumens. Thus, the consequent pressure gradient would always favor a HA-to-PV unidirectional flow of the blood or any fluid injectants, that is, APS via the PVP.

This study entailed the following methodological improvements. First, the applied bifunctional RIO contrast dye enabled high-quality images in DR, gross histology, and microscopy, allowing visualization and quantification of the liver vasculature [15, 16]. Secondly, the RIO/BSS were infused through catheterization of the carotid artery and under DR monitoring in order to get the whole-body angiography [14]. Third, the stepwise arterial infusion of a contrast dye in excised liver proved crucial to document the APS or HA-PVP-PV transition; otherwise only the image of the PV tree would appear as shown by Figures 3(c) and 4(f). Fourth, through our experiments, we noticed a similar behavior between RIO and BSS in hepatic microcirculation. Lastly, the “real-time” angiography suggested termination of only the PV, but unlikely HA, to the sinusoids.

## 5. Conclusions

By studying the intravascularly injected rat livers, we collected histomorphologic evidences including the following: (1) the PVP indeed exists between the lumens of the HA and PV, which is responsible to the APS of LPD that is injected from the HA but uncovered in the PV; (2) the intrahepatic vascular inflow appears only HA-PV unidirectional without an anatomic one-way valve; and (3) we proposed a biomechanical hypothesis by using fluid dynamics to extrapolate the observed virtual HA-PV one-way-valve phenomenon.

## Figures and Tables

**Table 1:** Semiquantitative assessment of histomorphological findings with BSS injections.

%	HA branches	PV branches	PVP	Sinusoids
HA injection	98	95	97	20
PV injection	6	98	55	90
*X* ^2^	169.55	0.59	48.36	98.99
*P* value	<0.001	>0.05	<0.001	<0.001

*Note*. BSS: barium sulfate suspension; HA: hepatic artery; PV: portal vein; PVP: peribiliary vascular plexus.

## Abbreviations

APS: Arterioportal shunt
BSS: Barium sulfate suspension
CBD: Common bile duct
CT: Computed tomography
DR: Digital radiography
HA: Hepatic artery
HBD: Hepatobiliary damage
LPD: Lipiodol
RIO: Red-stained iodized oil
PV: Portal vein
PVP: Peribiliary vascular plexus
TACE: Transarterial chemoembolization.
